# Risky Alcohol Drinking Pattern and Its Association with Educational Attainment and Wealth Index among Adult Men Population in Ethiopia: Further Analysis of 2016 Ethiopian Demographic Health Survey

**DOI:** 10.1155/2021/6646085

**Published:** 2021-04-14

**Authors:** Gedefaw Diress, Getinet Wondim

**Affiliations:** ^1^Department of Public Health, College of Health Sciences, Woldia University, Woldia, Ethiopia; ^2^Center for Disease Control Zonal Cordinator, Awi Zonal Health Department, Injibara, Ethiopia

## Abstract

Risky alcohol drinking is one of the major public health problems and an important health risk factor for premature death and disability worldwide. Identifying the determinants of risky alcohol drinking patterns is crucial for developing and improving intervention on drinking behavior. In Ethiopia, the role of educational attainment and affluence in reducing risky alcohol drinking patterns among men remains unclear. Therefore, this study aimed to assess the association of educational status and affluence with risky alcohol drinking patterns using national representative data in Ethiopia. Secondary data analysis was conducted on 12,688 adult men using data from the 2016 Ethiopia Demographic Health Survey (EDHS). The dependent variable was a risky alcohol drinking pattern which is defined as the consumption of alcohol every day in the last 12 months before the interview. Multivariable logistic regression was employed to assess the association between educational attainment, Ethiopian standard wealth index, and risky alcohol drinking pattern, after adjusting for the potential confounders. The overall magnitude of risky alcohol drinking patterns among men in Ethiopia was 4.5% (95% CI: 3.4–5.9). Of the total men who had ever taken alcohol, 9.7% of men drink almost every day in the last 12 months. The odds of having a risky alcohol drinking pattern were lower among men who completed secondary education (AOR = 0.56 (0.329–0.961)) and men who completed higher education levels (AOR = 0.35 (0.164–0.765)) as compared to men who did not attend any formal education. Adult men in the top two wealth quintiles were twice more likely to have risky alcohol drinking patterns than those in the lowest wealth quintile (AOR = 2.13 (1.254–3.605)). This study showed that from the total adult male population, nearly 5% of Ethiopian men had risky drinking patterns. Individuals with low educational status and greater affluence engaged in high-risk alcohol consumption behavior.

## 1. Introduction

Nowadays, alcohol consumption is one of the major public health problems and the third most important health risk factor for premature death and disability worldwide [1, 2]. It is associated with various chronic medical conditions and responsible for causing about 2.5 million deaths per year [1, 3–5] and a global loss of 139 million disability-adjusted life years [6].

In comparison to high-income countries, alcohol drinking is increasing and becoming more popular in Sub-Saharan Africa including Ethiopia. Studies conducted in different parts of Ethiopia showed that the magnitude of alcohol drinking has increased dramatically [7–9]. A recent nationwide study in Ethiopia revealed that nearly 25% of the population had a history of alcohol consumption [10]. Similarly, there is also a significant recent increment in the magnitude of risky alcohol drinking patterns in Ethiopia, particularly among men. Evidence from the very recent meta-analysis in the country showed that 9.0% of men aged 18–59 years engaged in hazardous or risky alcohol drinking pattern and behavior [10].

Many serious chronic diseases are linked to the quantity and frequency of alcohol consumption [11]. Therefore, identifying determinants related to drinking patterns (frequency) may be important for developing and improving intervention and treatment strategies. Among several determinants associated with risky alcohol drinking behaviors, socioeconomic status (educational attainment and wealth index) has been identified as a prominent risk factor [12, 13].

The frequency of alcohol consumption has been influenced by several factors. Among them, individuals' educational attainment and wealth index category play an important role in drinking patterns and behavior [14, 15]. Education is one of the major modifiable social determinants of alcohol drinking behavior. Previous studies, the majority carried out in the United States and Europe, have suggested that educational levels may influence alcohol drinking behaviors and patterns [16–19] but with conflicting results. Some studies [20, 21] showed that a higher level of education is associated with increased odds of reporting high-risk drinking (frequency of alcohol drinking). Conversely, few studies reported that a higher level of education is associated with reduced odds of reporting high-risk drinking [16, 22].

Affluence also tends to be associated with various risk or protective factors for many health-related problems. In the developed world, several studies revealed that individuals from lower socioeconomic status groups are more likely to have risky drinking behaviors [23–25]. In contrast, a study conducted in the United States revealed that higher income is associated with excessive alcohol consumption [26].

Although there are quite a lot of alcohol drinking prevalence studies in Ethiopia [7–9, 27, 28], evidence on the effect of education and wealth index (income) on risky alcohol drinking behaviors is limited. Furthermore, most of the previous studies were conducted in the specific geographic areas of Ethiopia [7–9, 27, 28] and mainly focus on urban areas [8, 28] which lacked national representation. Therefore, the objective of this study was to evaluate the effect of educational attainment and wealth index on risky alcohol drinking pattern and behavior using nationally representative Ethiopia Demographic and Health Survey (EDHS) data to inform policymakers.

## 2. Materials and Methods

This analysis used secondary data from the 2016 EDHS. It was a cross-sectional population-based study with two stages stratified sampling design. The 2016 Ethiopia Demographic and Health Survey (EDHS) is the fourth Demographic and Health Survey conducted in Ethiopia. The sampling frame used for the 2016 EDHS is the Ethiopia Population and Housing Census (PHC), which was conducted in 2007 by the Ethiopia Central Statistical Agency. The census frame is a complete list of 84,915 enumeration areas (EAs) created for the 2007 PHC. The 2016 EDHS sample was stratified and selected in two stages. Each region was stratified into urban and rural areas, yielding 21 sampling strata. Samples of EAs were selected independently in each stratum in two stages. Implicit stratification and proportional allocation were achieved at each of the lower administrative levels by sorting the sampling frame within each sampling stratum before sample selection, according to administrative units in different levels, and by using a probability proportional to size selection at the first stage of sampling. Data collection took place from January 18, 2016, to June 27, 2016. A detailed explanation of the methodology of 2016 EDHS is found somewhere else [30]. Data were obtained from the DHS program website: https://www.dhsprogram.com. In 2016 EDHS, 12,688 adult men have participated in the survey and all adult men were included in this analysis.

### 2.1. Study Population and Eligibility Criteria

All men aged 15–59 who were either permanent residents of the selected households or visitors who stayed in the household the night before the survey were eligible to be interviewed. Therefore, all men aged 15–59 years were the study population. In this study, we included only men aged 15–59 years who had a valid response to the next two questions during 2016 EDHS [1]. “Have you ever taken a drink that contains alcohol [2]? “How often you drank alcohol in the last 12 months before the survey?”

### 2.2. Study Variables

#### 2.2.1. Dependent Variable

The dependent variable was a risky alcohol drinking pattern. In the 2016 EDHS, survey respondents were asked two questions. [1] “Have you ever taken a drink that contains alcohol?” For this question, two response categories were included: yes or no. Survey respondents, who responded “yes” to the above question were also asked [2] “how often they drank alcohol in the last 12 months before the survey?” Regarding the frequency of alcohol consumption, four response categories were included: almost every day, at least once a week, less than once a week, and no in the last 12 months. These four groups were collapsed into two groups of nonrisky drinking behavior and risky drinking behavior. Risky alcohol drinking behavior was defined as the consumption of alcoholic beverages almost every day in the last 12 months before the interview. “Nonrisky drinking behavior” referred to those who have never drunk before (“lifetime abstainers”), those who drunk at least once a week or less than once a week in the last 12 months, and those who have not drunk in the past 12  months (“former drinkers”).

#### 2.2.2. Exposure Variables

In this study, the main exposure variables were educational status and wealth index. The participants' level of education was categorized into four groups: “no education,” “primary education,” “secondary education,” and “higher education level,” based on 2016 EDHS.

The wealth index is a composite measure of a household's cumulative living standard. The wealth index is calculated using easy-to-collect data on a household's ownership of selected assets, such as televisions and bicycles; materials used for housing construction; and types of water access and sanitation facilities [29]. In Ethiopia, the national wealth quintiles are compiled by assigning the household score to each usual household member, ranking each person in the household population by her or his score and then dividing the distribution into five equal categories (poorest, poor, middle, rich, and richest), each comprising 20% of the population. But, in the current study, we categorized the wealth index into three levels (poor, middle, and rich). The poor group consists of the poorest and poor wealth quantile. Similarly, rich and richest quantiles are combined into a rich group.

### 2.3. Covariates

The possible confounding variables include age (15–24, 25–34, 35–44, and 44–59 years), residence (urban and rural), occupation (working or not working outside the home at the time of the survey), marital status (married and not married), chat chewing media access, and number of children in the family (none, 1–4 children, and ≥5 children). Previous studies have identified the above variables as determinants of alcohol drinking. Therefore, these variables were selected a priori for inclusion in regression models as potential confounders.

Marital status was classified as married and nonmarried (single, divorced, and widow). Media exposure was classified based on response to how often respondents read a newspaper, listened to the radio, or watched television. Those who responded at least once a week to any of these sources were considered to have access to media.

### 2.4. Statistical Analysis

Participant characteristics were summarized using frequency and weighted percentage. Multicollinearity between independent variables was cheeked before fitting the final regression model. When two independent variables were found highly correlated, one was dropped.

Both educational status and wealth index were checked their association with risky alcohol drinking pattern behavior using bivariable logistic regression to estimate unadjusted odds ratios (ORs) and 95% confidence intervals (CIs). Thereafter, multivariable logistic regression models were used to calculate the adjusted ORs with 95% CI. In the multivariable model, the following variables were adjusted for age, religion, marital status, current job status, media exposure, chat chewing, and the number of children in the family.

The goodness of fit of the final logistic model was tested using the Hosmer–Lemeshow test at a *p* value of >0.05. Adjusted odds ratios with 95% CI were used to measure the association of level of educational status and wealth index with risky alcohol drinking pattern and behavior. All statistical techniques used a complex sampling design applied in the 2016 EDHS.

## 3. Results

### 3.1. Sociodemographic Characteristics of Men

In the current analysis, a total of 12,688 adult men were included. Of the total men who participated in this analysis, 35.1% of participants were in the age group of 15–24 years and 80.3% lived in a rural area. Almost 47% of men completed primary education level and 55.4% got married. Nearly half of the men were in the rich wealth index category. Almost a quarter of men had five and more children (Table 1).

### 3.2. Alcohol Drinking Behavior

**Figure 1:**
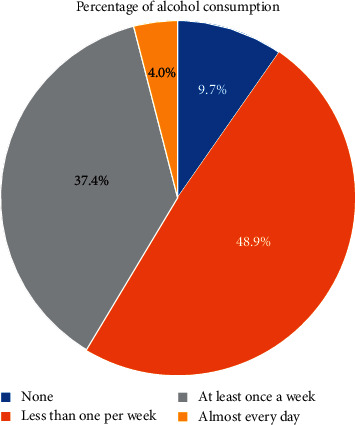
Frequency of alcohol consumption in the last 12 months among men who had ever taken a drink that contains alcohol in Ethiopia.

In this study, the weighted proportion of men who had risky drinking pattern was 4.5% (95% CI: 3.4–5.9). Of the total men who had ever taken alcohol, 9.7% of men drink almost every day in the last 12 months, 48.9% of men drink at least once a week, and 37.4% of men drink less than once a week (Figure 1).

**Figure 2:**
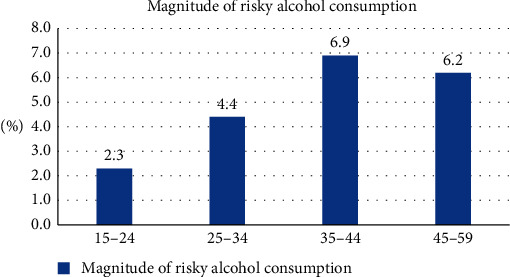
The magnitude of risky alcohol drinking patterns among men by age group, Ethiopia.

The magnitude of risky alcohol drinking patterns among men was varied among different age groups. Risky alcohol drinking behaviors develop gradually over the years. It was ranged from 2.3% in the 15–24 years age group to 6.9% in the 35–44 years age group (Figure 2).

#### 3.2.1. Association between Educational Attainment and Risky Alcohol Drinking Pattern

During bivariable logistic regression analysis, only the primary educational level was significantly associated with risky alcohol drinking patterns. But, at the multivariable‐adjusted model, both secondary educational status and higher educational level were significantly associated with risky alcohol drinking pattern behavior after adjusted for other independent variables. The odds of having risky alcohol drinking pattern behavior were 44% less likely among men who completed secondary education than men who did not attend any formal education (AOR = 0.56 (95% CI: 0.329–0.961)). Similarly, men who completed higher education levels were less likely to be risky alcohol consumers than men who did not attend any formal education (AOR = 0.35 (95% CI: 0.164–0.765)).

#### 3.2.2. Association between Wealth Index and Risky Alcohol Drinking Behavior

The higher wealth index quantile (rich category) was also significantly associated with risky drinking behavior in both unadjusted and adjusted analyses. Adult men in the rich wealth quintiles were two times more likely to have risky alcohol drinking patterns than those in the poor wealth quintile (AOR = 2.13 (1.254–3.605)) (Table 2).

## 4. Discussion

Risky alcohol drinking is a major public concern in Ethiopia. In the current study, nearly 5% of adult men had a risky drinking pattern. This finding is lower than a study conducted in rural India [30]. As expected, this study showed that risky alcohol drinking behavior increased among older age groups. It might be due to the fact that health problems or life events such as divorce, loss of family members, or unemployment were common among older peoples which might increase the risk of excessive alcohol drinking. The finding was similar to other studies [31, 32].

Socioeconomic status is one of the numerous factors determining alcohol drinking behavior in several parts of the world. Therefore, assessing the association of educational status and wealth index with risky alcohol drinking behavior is essential for evidence-based public health intervention in Ethiopia. The finding of the current study revealed that higher educational status has a positive significant effect on the reduction of risky drinking behavior. The finding is comparable with previous studies conducted in different parts of the world [33, 34]. Education has an important role in changing lifestyles, behaviors, social networks, and social norms. Education increases individuals' chance to access information on the adverse effect of excessive alcohol use [35]. As a result, educated persons may prefer healthy lifestyle habits and avoid risky behavior like excessive alcohol consumption [36, 37]. However, the finding of this study is inconsistent with previous studies that reported higher educational attainment which increased alcohol intake frequency and the odds of risky drinking behavior [38–40]. The difference might be mainly due to the variation in the population culture and in different countries.

People in the lowest wealth index group, due to unequal access to health care, are prone to several risky behaviors such as smoking, substance use, and alcohol drinking [41]. However, in this study, a higher wealth index was positively associated with risky drinking behavior which is consistent with the findings of other studies [25, 38, 39, 42]. This might be due to the fact that men in rich wealth index quantile can afford the price of alcoholic beverages which might increase the risk of alcohol consumption [43, 44]. But few studies showed that low income was associated with higher odds of problem in drinking [45, 46].

The current study has several strengths including a large nationally representative sample size, availability of detailed data on confounders, and standardized, high-quality data collection tools. However, there are several limitations to consider. First, as cross-sectional data were used, we cannot assign causality. Second, estimation of alcohol consumption in 2016 EDHS was entirely on self-reporting which might result in social desirability biases and may have been subjected to under-reporting. Some people who drink small amounts of alcohol may report their intake as none that results in the odds ratio being systematically low.

## 5. Conclusion

Both educational status and wealth index were associated with risky alcohol drinking patterns, which must be considered when designing preventative interventions. Individuals with low educational status and higher wealth index quantile were engaged in more frequent and excessive drinking behavior. Future strategies to reduce risky drinking patterns should target those in rich wealth quantile and policymakers should also focus on individual educational attainment. Future studies should consider factors at the neighborhood level (such as social interaction) and community level.

## Figures and Tables

**Table 1:** Sociodemographic characteristics of the study sample (*N* = 12,688).

Variables	Categories	Numbers	Weighted %
Age	15–24	4502	35.1
	25–34	3615	28.5
	35–44	2592	20.4
	45–59	1979	16.0

Residence	Urban	3866	19.7
	Rural	8822	80.3

Religion	Orthodox	5577	44.8
	Protestant	2110	21.7
	Muslim	4866	31.4
	Others	2352	2.1

Educational status	No education	3517	30.3
	Primary	5331	46.5
	Secondary	2233	14.6
	Higher	1607	8.7

Current job status	Not working	2093	11.3
	Working	10595	88.7

Marital status	Not married	5680	44.5
	Married	7008	55.4

Wealth index	Poor	4718	34.1
	Middle	1739	19.3
	Rich	6231	46.6

Number of children	No child	5873	44.5
	1–4 children	3754	29.7
	≥5 children	3061	25.8

Have you ever chewed chat	Yes	3895	27.1
	No	8631	72.9

**Table 2:** Association of educational attainment and wealth index with risky alcohol drinking pattern among adult men in Ethiopia, (*N* = 12,688).

Variable		Risky alcohol drinking pattern		
		OR (95% CI)		*p* value
		Unadjusted analysis	Adjusted analysis^b^	
Educational status	No education	1	1	
	Primary	0.59 (0.427–0.808)	0.82 (0.593–1.138)	0.147
	Secondary	0.64 (0.380–1.088)	0.56 (0.329–0.961)	0.016
	Higher	0.65 (0.336–1.237)	0.35 (0.164–0.765)	0.008

Wealth index	Poor	Ref.	Ref.	
	Middle	1.45 (0.977–2.142)	1.39 (0.932–2.067)	0.106
	Rich	2.28 (1.472–3.536)	2.13 (1.254–3.605)	0.005

AOR, adjusted odds ratio; COR, crude odds ratio; CI, confidence interval. *Note*. ^b^Adjusted for age, residence, religion, marital status, current working status, media exposure, and chat chewing.

## Data Availability

The data are available from the corresponding author on a reasonable request.

## Abbreviations

AOR: Adjusted odds ratio
COR: Crude odds ratio
CI: Confidence interval
SSA: Sub-Saharan Africa.
